# Different predictors of intimate partner and natal family violence against women

**DOI:** 10.1093/emph/eoac019

**Published:** 2022-05-02

**Authors:** Olympia L K Campbell, Ruth Mace

**Affiliations:** Department of Anthropology, University College London, London WC1H 0BW, UK

**Keywords:** violence against women, Jordan, cousin marriage, sexual conflict, parents–offspring conflict

## Abstract

**Background:**

Violence against women is often studied in the context of violence from intimate partners. However, women receive violence from a wider range of individuals—such as their natal kin—including their siblings, parents, uncles and cousins. Applying insights from evolutionary theory, we examine whether cousin marriage, which has been hypothesized to both reduce the risk of partner violence but increase the risk of natal family violence, associates differently with each type of violence. Second, we test whether common risk factors for partner violence, such as wealth, associate similarly with natal violence.

**Methodology:**

We analyse over 16 000 Jordanian women from three cohorts of the Jordan Demographic Health Surveys. Predictor variables include type of cousin marriage (patrilateral or matrilateral), education, wealth, number of children, urban living and polygyny. Outcome variables include whether a woman’s husband or her natal family has ever been physically violent towards her.

**Results:**

Being married to a patrilateral cousin but not a matrilateral cousin is associated with a reduced risk of reporting intimate partner violence (IPV). By contrast being married to a matrilateral cousin but not a patrilateral one is associated with a reduced risk of reporting natal family violence. As expected, wealth is negatively associated with reporting partner violence, but we find no association with reports of natal family violence. Finally, individuals with more children are more likely to report IPV.

**Conclusions and implications:**

Findings indicate the importance of distinguishing between types of cousin marriage and highlight substantial differences in risk factors for intimate partner compared to natal family violence.

**Lay Summary:**

Sociodemographic risk factors, such as wealth, may associate differently with intimate partner and natal family violence. Results suggest that whether cousin marriage is protective of violence may depend on the type of cousin and secondly, that violence can have fitness relevant outcomes.

## BACKGROUND

Violence against women (VAW) is often studied in the context of either intimate partner violence (IPV) or sexual violence by non-intimate partners. However, natal family violence is also common, for example in honour cultures, women may receive violence from their parents, siblings, uncles and cousins—among others [1]. It is not known whether the risk factors for violence from natal kin are similar to those for IPV. Previous literature has highlighted cousin marriage as being protective of IPV [2], whereas a separate literature has argued that it may be a risk factor for natal family violence [3]. VAW is less commonly examined from an evolutionary perspective, which predicts that violence may be indicative of an underlying evolutionary conflict of interest, and generates novel predictions based on evolutionary theory.

When viewed through an evolutionary lens, IPV is considered an outcome of sexual conflict. Sexual conflict occurs when a man can increase his reproductive fitness via a behavioural ‘tool’, such as IPV, capable of influencing female behaviour, but which comes at a cost to the woman’s fitness, or vice versa [4]. IPV is often considered a mate guarding behaviour that serves to increase or maintain exclusive sexual access to a woman through preventing female adultery, preventing women from leaving the relationship, increasing sexual access to said woman and overall increasing her deference to her husband’s fitness-relevant objectives [5–8]. Sexual conflict theory predicts several IPV patterns that are observed: younger women, who are more fertile, are at greater risk [9]; wealthier and more educated individuals—who in the case of men have recourse to other forms of mate retention, such as greater resources, and in the case of women greater bargaining power—are at lower risk [9]; and women married polygynously, who are likely to have higher levels of conflict with their husband and co-wives over resources, are at higher risk [10]. Similarly, cues of infidelity or separation often trigger IPV [6], such as if a husband does not know where their partner is or, where female employment is uncommon and gender segregation common, if women work outside the home or alongside male colleagues [11].

Cross-culturally women who report IPV have more children [12], in line with IPV having fitness relevant outcomes, although important confounders such as age at marriage and socioeconomic status are often not controlled for. However, one study on a horticultural population in Bolivia, found that reporting a major IPV incident was associated with a significantly increased likelihood of birth within the following year, indicating a causal relationship [7].

In terms of natal family violence, not much has been said by evolutionary scientists, but there is a recognition within evolutionary theory that there can be conflicts of interest between parents and offspring and between siblings. Parent–offspring conflict [13] results from a divergence between the fitness-enhancing aims of parents’ vis-à-vis their offspring and can lead to violence as a means to mould the behaviour of offspring towards behaviours that maximize parental fitness [14]. Parent–offspring conflict commonly arises over mating preferences, and humans appear to be unique in the animal kingdom in the ability of parents to control or influence the mating of their children [15]. Parents and offspring may disagree over the relative value of different qualities in a partner. For example, whilst genetic benefits are transmitted only to the offspring of the couple, material benefits that an individual brings to a marriage can be transmitted to affinal kin of their spouse. Thus, it is predicted that on average parents prioritize in a child’s partner what will be beneficial for the wider family [16], whereas offspring prefer qualities associated with genetic quality, such as good looks and physical strength [17]. Furthermore, mothers and fathers, or wider matrikin and patrikin, may also disagree over the value of material versus genetic benefits if material benefits flow unequally to patrikin compared to matrikin [18], which indeed they would in groups structured around patrilineal descent and inheritance, such as Jordan [19].

A societal preference for cousin marriage has been hypothesized to be associated with the risk of violence from both intimate partners and natal family. Cousin marriage may be associated with a reduced risk of IPV as women are better acquainted with their husband and more likely to reside near their natal home and be supported by kin [2]. By contrast, others have argued that cousin marriage is associated with increased violence from natal kin, often in the context of honour-related violence [3, 20, 21], which could reflect parent–offspring or sibling conflict over said marriages. Why there might be parent–offspring conflict over cousin marriage has not been examined. Cousin marriages can help consolidate wealth within families [22, 23] and aid in building tight kinship networks [24], which may be indispensable in societies where kin support is essential to survival. Cousin marriage also reduces bride price and dowry payments [25, 26]. By contrast, offspring suffer the cost of inbreeding depression [27]. Whilst material benefits from cousin marriage are likely to be shared between the couple, parents and the extended family, genetic costs will be borne heavily by the consanguineous couple. Of course, parents also suffer this genetic cost, as they are related to their grandchildren, but it is unlikely that all offspring of a couple will marry consanguineously. Thus, whilst parents suffer the cost of inbreeding depression in one set of grandchildren, this is offset by the fact that their other offspring will likely outbreed, thus allowing parents to reap the material or social benefits of marrying some of their children consanguineously, whilst also reaping the genetic benefits of exogamous marriage in the rest of their children.

**Figure 1.**
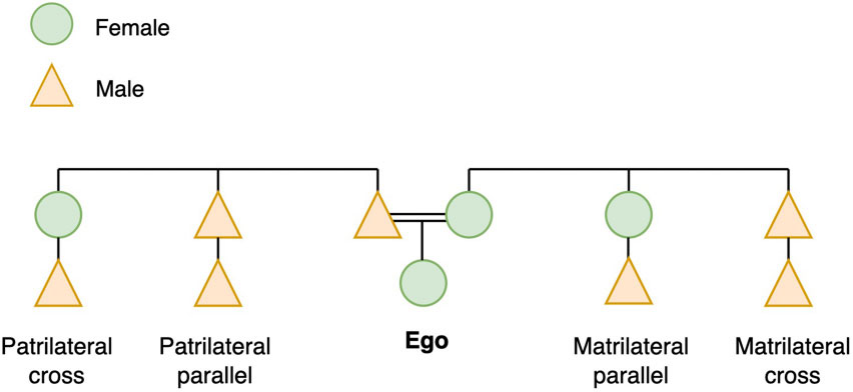
Types of cousins in relation to ego

Much of the literature does not differentiate between different types of cousin marriage, such as whether it is a patrilateral (on the father’s side), matrilateral (on the mother’s side), cross (offspring of a parents’ opposite sex sibling) or parallel cousin (offspring of a parents’ same sex sibling) (Fig. 1). Evolutionary theory would expect different associations with violence, depending on the type of cousin marriage. For example, behavioural ecologists have argued that women are more at risk of IPV in patrilocal societies, where post-marital residence is with the husband and his relatives, due to women being separated from both female kin, with whom she could form coalitions, but also male kin who might defend her [28]. However, post-marital residence may change depending on whether one marries consanguineously or not. For example, in a patrilocal system, marrying a matrilateral cousin should lead to a greater distance of dispersal than if you married a patrilateral parallel cousin (father’s brother’s son), as men should be residing close to their brothers, whereas women are less likely to be residing close to their siblings. Thus, we may only see a protective association between cousin marriage and IPV in patrilateral parallel cousin marriages.

Whether there may be increased parent–offspring conflict between different types of cousin marriage has not been examined. It is unlikely that the inbreeding cost will differ on average between ego and the different types of first cousins even if there is a preference for a particular kind of cousin marriage, such as patrilateral parallel cousin marriage, as every individual is a member of both a matriline and a patriline, and their patrilines should carry the same average inbreeding load. However, other costs to daughters of marrying consanguineously may vary between cousin types. In Jordan, descent is patrilineal and, at least historically, extended families, households and clans were broadly structured on blood ties between agnates, with agnatic kin being heavily relied upon for support, whether financial or social [19]. The preferred form of marriage in this kinship system, common in Jordan and amongst other Arab populations, is between patrilateral parallel cousins [29, 30], as this consolidates patrilineal kin groups and allows inheritance, or any wealth transfers at marriage, to remain within the patriline [24]. With respect to women, this results in them remaining under the control of their male agnates and subordinates their interests to the agnatic group. Costs associated with this may be reduced female autonomy and freedom to make optimal decisions, such as those relating to reproductive timing. For example, consanguineously married couples tend to have more offspring although this is largely explained by earlier age at first birth and faster replacement of infants that have died [31], which likely has a significant physical cost to women. There is also evidence that fertility benefits of consanguineous marriage are offset by reduced fertility in the following generation [32].

Thus, where there is a preference for patrilateral parallel cousin marriages, one might see the intensity of parent–offspring conflict increase with each passing generation of said cousin marriage, as the benefits that accrue to parents from wealth and kin network consolidation might increase, whilst the costs to offspring from inbreeding depression and patrilineal control also increase. Additionally, if benefits from cousin marriage are reaped mostly by the patriline, then brothers may also have a stronger vested interest in the marriage choices of their siblings, increasing sibling conflict.

The association between cousin marriage and IPV is inconsistent, with some studies finding that cousin marriage is protective [33] or that being separated from male kin increases vulnerability to violence [34], others that cousin marriage is associated with increased IPV [26], and some finding no association [35, 36]. Women in focus groups have highlighted that cousin marriages are preferential due to the danger and uncertainty of marrying an unrelated individual [34, 37]. Additionally, consanguineously married women have been found to be more likely to believe that husbands are justified in beating their wives [38], while others find no association [39]. In terms of natal family violence, cousin marriage was associated with an increased risk of reporting honour-related violence, but only where the marriage was forced or fully arranged [21]. Whether these associations change based on the type of cousin marriage is less known.

This paper has two aims. First, we examine whether the risk factors for reported IPV and natal family violence differ, with a particular focus on cousin marriage. Second, we examine whether differences in risk factors fit with predictions derived from evolutionary behavioural ecology. We propose three hypotheses:

1. Women married consanguineously will report less IPV compared to those married to unrelated individuals, and those married patrilaterally will report less IPV than those married matrilaterally.
2. Women married consanguineously will report more natal family violence than those married to unrelated individuals, and those married patrilaterally will report more natal family violence than those married matrilaterally.
3. Women who report violence will have more children.

Additionally, we explore whether individual level risk factors such as education, wealth and polygyny correlate similarly across both types of violence.

## METHODOLOGY

### Participants

Data is from the 2007, 2012 and 2017 Jordan Demographic and Health Surveys (DHS) [40]. The Jordan DHS is stratified by 12 governate regions and between urban and rural areas. One ever-married woman aged 15–49 years is selected randomly from each household to complete the domestic violence module. A total of 17 323 women over the three cohorts were surveyed in the domestic violence module. Further information on the sample design can be found in the final reports.

### Outcome variables

Women were asked if their current (or last) husband had ever been physically violent towards them. Violence included being pushed, had something thrown at, slapped, punched, hit with something, having their arm twisted or hair pulled, being kicked, dragged, strangled, burnt and being threatened with a weapon. Second, women were asked ‘from the time you were 15 years old has anyone other than your (last) husband hit, slapped, kicked, or done anything else to hurt you physically?’ If yes, respondents were asked who had hurt them in that way, and answers included mother, father, brother, sister. Third, women were asked about whether husbands were ever justified in beating their wives in several situations, including if a wife went out without telling her husband; neglected the children; argued with her husband; disobeyed her husband; or burned the food. Questionnaires can be found in the DHS final reports.

Three binary variables were created where individuals were given a score of 1 if they had: (i) reported violence from their husband; (ii) reported violence from a related family member (mother, father, sister or brother); and (iii) justified a husband’s abuse.

### Covariates

Socioeconomic status was measured using the DHS-derived wealth index and education was measured by the highest level of school the individual attended. Other variables included employment status, total number of children, age at marriage, age at survey, year of birth, survey year and whether their husband currently had any other wives besides themselves. Respondents were asked whether they were related to their husband and if so what kind of relation it was. Women were classified as being married to (i) an unrelated individual, (ii) a patrilateral cousin and (iii) a matrilateral cousin. Those married to a double first, patrilateral parallel, patrilateral cross or patrilateral second cousin were classified as patrilateral. Double first cousins occur when two siblings from one family marry two siblings from another family and the resulting offspring share both sets of grandparents (Supplementary Fig. S1). These individuals are related to each other both matrilaterally and patrilaterally but since we are hypothesizing that patrilateral marriages are protective of IPV, are classified within the patrilateral group.

A second seven-category variable was created where women were classified as married to (i) a double first cousin who she is related to via both her fathers’ brother and her mothers’ sister, (ii) a double first cousin related via both her fathers’ sister and mothers’ brother, (iii) a patrilateral parallel cousin, (iv) all other patrilateral relatives (patrilateral cross-cousin and second cousin), (v) a matrilateral parallel cousin, (vi) all other matrilateral relatives (matrilateral cross-cousin and second cousin) and (vii) an unrelated individual; 149 individuals who were more distantly related to their husband than second cousins were also classified as unrelated.

### Analysis

Multi-level logistic regressions with random intercepts for region were used to investigate the difference in associations between covariates and IPV, natal family violence and justification for violence. Age at the time of survey was controlled for and grand-mean centred to address convergence issues. An interaction term was also included to assess whether the association between cousin marriage and violence differed by survey year. Univariate analyses between cousin marriage and the two types of violence are presented in the Supplementary Materials (Supplementary Tables S3 and S4).

## RESULTS

Between those surveyed in 2007 and 2017 reported IPV declined from 19.60% to 14.83% and reported natal family violence declined from 16.41% to 5% (Table 1). Most reported violence from natal family members was perpetrated by male family members with 4.98% and 5.67% of women reporting violence from father’s and brothers, respectively, although mothers were also common perpetrators. The percentage of women who thought violence from husbands was justified in at least one case also fell from 76.66% of women to 26.77%.

**Table 1.** Percentage and raw numbers of women who reported violence from husbands and natal family members or justified violence by survey year.

	Violence from husband % (*n*)	Violence from natal family % (*n*)					Justification of violence % (*n*)
		Mother	Father	Sister	Brother	Any natal family member^a^	
**2007**	19.60	7.14	6.97	1.48	7.26	16.41	76.66
	(675)	(246)	(240)	(51)	(250)	(565)	(2640)
**2012**	19.44	4.50	6.33	0.81	7.98	15.17	44.84
	(1366)	(316)	(444)	(64)	(560)	(1065)	(3151)
**2017**	14.83	1.58	2.52	0.20	2.40	5.00	26.77
	(1016)	(105)	(167)	(13)	(159)	(332)	(1834)
**Total**	**17.65**	**3.90**	**4.98**	**0.74**	**5.67**	**11.47**	**44.02**
	**(3057)**	**(667)**	**(851)**	**(128)**	**(969)**	**(1962)**	**(7625)**

a This column is the total % of women who received violence from any natal family member and not the sum of cases. Bold represents total values.

**Table 2.** Percentage and raw numbers of women married to a blood relative by consanguinity type and survey year

	Double first cousins	Patrilateral parallel cousin	Patrilateral cross cousin	Patrilateral second cousin	Matrilateral parallel cousin	Matrilateral cross cousin	Matrilateral second cousin	Unrelated
2007	4.20	10.43	4.15	10.35	4.94	3.29	4.76	57.88 (6295)
	(457)	(1134)	(451)	(1126)	(537)	(358)	(518)	
2012	1.52	9.28	4.88	8.90	5.69	3.37	3.38	62.98
	(172)	(1054)	(554)	(1010)	(646)	(382)	(384)	(7150)
2017	3.86	6.84	3.04	5.48	3.48	2.48	2.75	72.08
	(567)	(1004)	(446)	(805)	(511)	(364)	(404)	(10588)
**Total**	**3.24**	**8.65**	**3.93**	**7.97**	**4.59**	**2.99**	**3.54**	**65.10**
	**(1196)**	**(3192)**	**(1451)**	**(2941)**	**(1694)**	**(1104)**	**(1306)**	**(24033)**

These numbers are derived from the entire sample of women surveyed, not just those who completed the domestic violence module. Bold represents total values.

Of the 34.9% of women who were married consanguineously, there is a clear preference for patrilateral relatives, particularly patrilateral parallel cousins and patrilateral second cousins with 8.65% and 7.97% of women being married in this way, respectively (Table 2). Consanguinity (second cousin or closer) fell over the three surveys from 42.12% in 2007 to 27.92% in 2017 and the largest declines were in marriages between patrilateral parallel cousins and patrilateral second cousins, which fell by 3.59% and 4.87%, respectively.

### Do individuals married consanguineously report less IPV?

**Figure 2.**
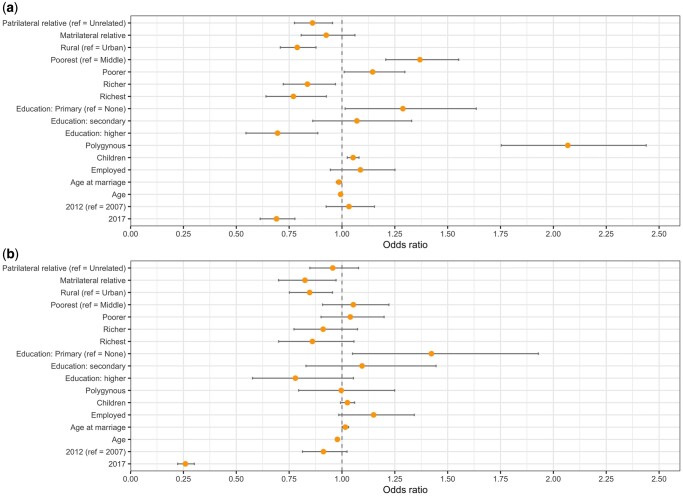
Odds ratios and confidence intervals from multi-level logistic regressions of the likelihood of (a) reporting violence from a husband and (b) reporting violence from a natal family member, splitting consanguinity into patrilateral and matrilateral relatives

Overall, women married consanguineously were less likely to report IPV; however, the magnitude of this association differed depending on whether the husband was related on their father or their mother’s side (Fig. 2, Table 3). Being married to a patrilateral relative was significantly associated with a 14% reduction (OR = 0.86, 95% CI [0.78–0.96]) in reporting IPV, compared to being married to an unrelated individual. Being married to a matrilateral relative was not significantly associated with the likelihood of reporting IPV although it trended in a negative direction (OR = 0.93, 95% CI [0.81–1.06]).

**Table 3.** Odds ratios (ORs) and confidence intervals (CIs) of multi-level logistic regressions

	Model A: Violence from husband	Model B: Violence from natal family
	OR (95% CI)	OR (95% CI)
Ref: Unrelated	0.86^**^	0.96
**Patrilateral relative**	(0.78–0.96)	(0.85–1.08)
**Matrilateral relative**	0.93	0.82^*^
	(0.81–1.06)	(0.70–0.97)
Ref: Urban	0.79***	0.85^*^
**Rural**	(0.71–0.88)	(0.75–0.96)
Ref: Middle	1.37***	1.05
**Poorest**	(1.21–1.55)	(0.91–1.22)
**Poorer**	1.15*	1.04
	(1.01–1.30)	(0.90–1.20)
**Richer**	0.84*	0.91
	(0.72–0.97)	(0.77–1.07)
**Richest**	0.77**	0.86
	(0.64–0.93)	(0.70–1.06)
Ref: No education	1.29*	1.42^*^
**Primary**	(1.01–1.64)	(1.05–1.93)
**Secondary**	1.07	1.09
	(0.86–1.33)	(0.83–1.45)
**Higher**	0.70**	0.78
	(0.55–0.89)	(0.58–1.05)
**Polygynous**	2.07***	1.00
	(1.75–2.44)	(0.79–1.25)
**Children**	1.05***	1.03
	(1.03–1.08)	(0.99–1.06)
**Employed**	1.09	1.15
	(0.94–1.25)	(0.98–1.34)
**Age at marriage**	0.99*	1.02^*^
	(0.97–1.00)	(1.00–1.03)
**Age**	0.99	0.98^***^
	(0.99–1.00)	(0.97–0.99)
Ref: 2007	1.03	0.91
**2012**	(0.93–1.15)	(0.81–1.02)
**2017**	0.69***	0.26^***^
	(0.61–0.78)	(0.22–0.30)

Model A considers the likelihood of reporting violence from a husband, Model B the likelihood of reporting violence from a natal family member.

* *P* < 0.05;

** *P* < 0.01;

*** *P* < 0.001.

**Figure 3.**
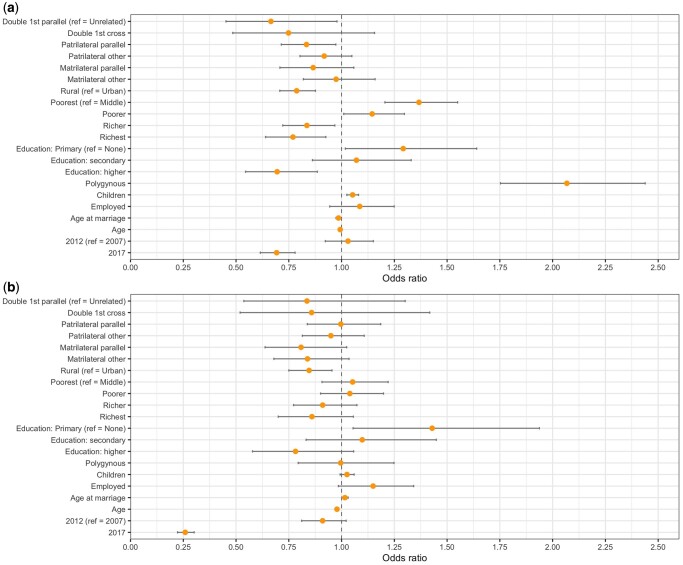
Odds ratios and confidence intervals from multi-level logistic regressions of the likelihood of (a) reporting violence from a husband and (b) reporting violence from a natal family member, splitting consanguinity down into further constituent types

Breaking consanguinity down further into its constituent types yielded further differences (Fig. 3, Supplementary Table S1). Being married to a double first cousin, where women were related to their husbands via their father’s brother (and their mother’s sister), or a patrilateral parallel cousin was associated with a 33% (OR = 0.66, 95% CI [0.45–0.98]) and 17% (OR = 0.83, 95% CI [0.71–0.97]) reduction in the odds of reporting IPV compared to unrelated marriages, respectively. By contrast, no other type of cousin marriage was significantly associated with the odds of reporting IPV, compared to unrelated marriages, although all trended in the negative direction.

Despite consanguinity being associated with a reduced likelihood of reporting IPV, the association with the likelihood of justifying violence from a husband trended in a positive direction (Supplementary Fig. S2 and Table S2) and narrowly missed significance for women married to patrilateral relatives (OR = 1.07, 95% CI [0.99–1.18]).

### Do individuals married consanguineously report more natal family violence?

Individuals married consanguineously were less likely to report natal family violence (Fig. 2, Table 3), although this was only significant for matrilateral cousins who were 18% less likely to report violence (OR = 0.82, 95% CI [0.70–0.97]), compared to those in unrelated marriages. Breaking consanguinity down further for natal family violence yielded no significant associations (Fig. 3, Supplementary Table S1) although odds ratios for matrilateral marriages were lower than those for patrilateral marriages.

However, introducing an interaction term between type of consanguinity and survey year showed a positive association between patrilateral parallel cousin marriage and both kinds of violence (Supplementary Table S3), but particularly for natal family violence, for the 2017 cohort (OR = 1.82, 95% CI [1.11–2.97]). In 2017, despite overall reporting less violence, individuals married to said cousin were 68% more likely to report natal family violence and 4% more likely to report IPV, relative to women married to unrelated individuals.

### Do women who report violence have more children?

**Figure 4.**
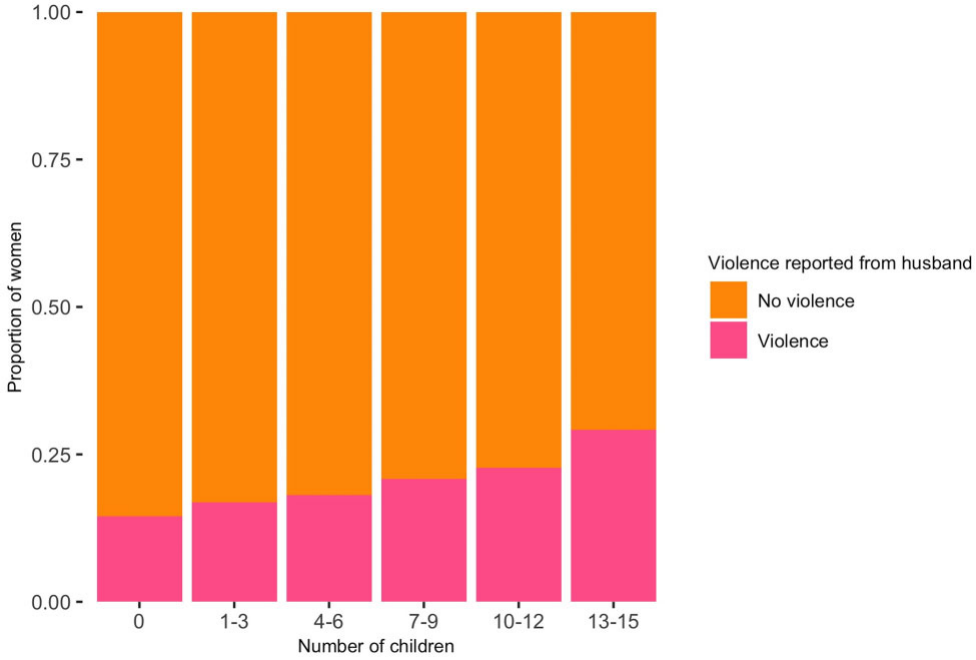
Proportion of women who reported violence from their husband grouped by number of children

Women with more children were more likely to report IPV; with individuals being 5% more likely (OR = 1.05, 95% CI [1.03–1.08]) to report IPV with each additional child (Fig. 4) but was not significantly associated with natal family violence. Women married consanguineously also had more children (Supplementary Fig. S3).

### Do other risk factors differ between IPV and natal family violence?

Associations with rural residence, education, employment and survey year were similar across IPV and natal family violence (Fig. 2, Table 3). Individuals living in rural areas compared to urban areas were 21% (OR = 0.79, 95% CI [0.71–0.88]) and 15% (OR = 0.85, 95% CI [0.75–0.96]) less likely to report IPV and natal family violence, respectively. Individuals with primary education compared to no education were 29% (OR = 1.29, 95% CI [1.01–1.64]) and 42% (OR = 1.42, 95% CI [1.05–1.93]) more likely to report IPV and natal family violence, respectively. Comparably, women with higher education were 30% (OR = 0.70, 95% CI [0.55–0.89]) and 22% (OR = 0.78, 95% CI [0.58–1.05]) less likely to report IPV and natal family violence, respectively, although this was not significant for natal family violence. Being employed was not significantly associated with the likelihood of reporting either type of violence although it trended towards a positive association for both. Survey year had a large association with the likelihood of reporting violence, with individuals surveyed in 2017 compared to 2007 being 30% (OR = 0.69, 95% CI [0.61–0.78]) and 74% (OR = 0.26, 95% CI [0.22–0.30]) less likely to report IPV and natal family violence, respectively. Similarly, women surveyed in 2017 were 89% (OR = 0.11, 95% CI [0.10–0.12]) less likely to justify violence from their husbands, compared to women surveyed in 2007 (Supplementary Fig. S2).

Wealth and polygyny associated differently with IPV and natal family violence (Fig. 2, Table 3). Wealth was strongly and significantly negatively associated with reported IPV, with individuals in the poorest quintile being 37% more likely (OR = 1.37, 95% CI [1.21–1.55]) and those in the richest quintile being 23% (OR = 1.23, 95% CI [0.64–0.77]) less likely to report IPV compared with those in the middle quintile. By contrast, although the odds ratios trended in a similar direction wealth was not significantly associated with reported natal family violence. Individuals married polygynously were over twice as likely (OR = 2.07, 95% CI [1.75–2.44]) to report IPV compared to a woman in a monogamous marriage but was not associated with reporting natal family violence.

## CONCLUSIONS

Being married consanguineously was associated with a reduced likelihood of reporting violence from a husband, but not from a family member, consistent with theory and empirical work showing consanguinity is protective of IPV [2, 25, 41]. That women married to their patrilateral relatives had a stronger negative association with IPV supports behavioural ecology predictions that women residing near kin will have greater status. Indeed, it is striking that the only two subtypes of cousin marriage that were statistically significantly associated with IPV was the double first parallel cousin marriage, which means women are related to their husbands through their father’s brother (Supplementary Fig. S1), and a patrilateral parallel cousin marriage. These are the two types which, if post-marital residence is with the husband’s family, should result in the woman residing near her patrilateral male kin, particularly members of her father’s patrilineage. Indeed, in focus groups in Jordan, women highlighted being separated from their male kin as a contributing factor to their risk of IPV [34].

It is also possible that women in consanguineous unions are less likely to report IPV, either because they are more likely to engage in conflict avoidance strategies, or because of potentially higher repercussions of damaging the family dynamic were their indiscretion to be revealed. Qualitative research in Egypt, Pakistan and Bangladesh has shown that women married to their relatives felt obligated to tolerate higher levels of mistreatment than women married to unrelated individuals, due to the risk of damaging relationships with kin [37, 38, 42]. In Bangladesh, women agreed that patrilateral parallel cousin marriages were particularly tense, whereas matrilateral parallel cousins were preferential, due to relations between sisters being more relaxed than those between brothers. Sisters often reside in different places and have no common property to argue over, whereas disputes about inheritance in the patrilineage are common [37]. Women also expressed the desire to marry unrelated individuals and be less dependent on kin who might make unreasonable demands, particularly patrilineal kin [37]. Whilst not statistically significant, the association between consanguinity and justification of husband violence trended in a positive direction, particularly for patrilateral parallel cousins, somewhat consistent with trends found in Egypt [38] and suggestive of more conservative values around gender equality within consanguineous marriages.

That being married to a relative was not associated with an increased risk of reporting violence from natal family members compared to unrelated married couples, does not support the hypothesis that cousin marriage in general produces high levels of parent–offspring conflict. However, we do find some evidence of possible variation in parent–offspring conflict between types of cousin marriage, with those married to a matrilateral cousin being significantly less likely to report natal family violence, whereas being married to a patrilateral parallel cousin carried the same risk of reporting natal family violence as being married to an unrelated individual. If natal family violence does capture some degree of parent–offspring or sibling conflict over marriage, then this result indicates that matrilateral cousins may be the preferred marriage partner by offspring, whereas patrilateral parallel cousins and unrelated individuals are the least desired. As discussed, it may be that in societies with a longstanding preference for patrilateral parallel cousin marriage, parent–offspring conflict is more intense due to a ratcheting effect over generations of both the benefits that accrue to parents and wider kin, but particularly the costs that offspring suffer.

In Jordan, whilst many couples become somewhat acquainted independently of family, most marriages are in some respects arranged, and how long couples are allowed to get to know each other for varies greatly [43–45]. Often women have more interaction with their extended kin due to the limited number of respectable places that unrelated men and women can meet, although this is changing rapidly through changes to society, such as increasing numbers of Jordanian women going into higher education [43]. In Bangladesh, the rise in ‘love marriages’ that are replacing more strictly arranged marriages has contributed to an increase in matrilateral cousin marriage, which appears to be the preferred choice given the available pool of men that women interact with [37]. Thus, while marrying a patrilateral parallel cousin may carry higher risks of inbreeding depression and result in women being reliant on and controlled by their male relatives, marrying an unrelated individual can also be risky as couples are usually less well acquainted, marriage negotiations can be more complicated and the bride may be less well treated by her in-laws [25, 26, 42, 46], leaving matrilateral cousins as the most desired balance of risks.

That individuals surveyed in 2017 were much less likely to report both forms of violence and to justify violence indicates that norms around violence have shifted considerably since 2007. In 2017 and 2018, a number of legal changes were made, including the repealing of Article 340 of the penal code of Jordan that allowed reduced sentences for men who murdered their female relatives on the basis of them committing adultery. This normative change around women’s rights could lead to particularly tense parent–offspring conflict around marriages deemed more conservative by young people, such as patrilateral cousin marriages. If so, we would expect the association between patrilateral parallel cousin marriage and natal family violence to differ by survey year. Indeed, introducing an interaction term between survey year and type of cousin marriage indicated that within the 2017 sample, patrilateral parallel cousin marriage is positively associated with risk of natal family violence relative to unrelated marriage.

Having more children was associated with increased risk of reporting IPV consistent with the hypothesis that IPV increases reproductive success potentially through increasing sexual access to a wife or reducing the age at first birth [7, 12]. It is well known that men often express less desire to limit childbearing, as is the case for Jordan [40]. To our knowledge, this is one of the first studies to document an association between IPV and number of offspring in a non-natural fertility population whilst also controlling for major confounders like wealth, education and age at marriage.

Polygyny was found to be a strong predictor of IPV, consistent with other literature [10]. Polygyny can be the outcome of sexual conflict where males benefit reproductively from polygyny, whereas women suffer a cost, such as lower fertility or higher child mortality, as has been found in the Dogon of Mali [47]. Additionally, competition and conflict between co-wives and wives and husbands over resources can lead to violence [48].

Increased wealth was associated with reduced odds of reporting IPV, consistent with most other literature [49]. Interestingly, however, wealth was not significantly associated with natal family violence. If natal family violence reflects parent–offspring conflict over marriage, or female behaviour in general, then one would expect VAW to occur irrespective of family wealth as wealthy families also control the behaviour of their female kin—indeed the reputational costs for wealthy families may be greater. Furthermore, primary education was associated with increased risk of both forms of violence perhaps implying that violence is used to reassert dominance against the growing independence of educated women, also reflected by women being less likely to justify violence the more educated they are. This trend reverses with higher education, indicating that there could be a U-shaped relationship between VAW and education (or female emancipation generally), with women receiving less violence at very low and very high levels of education.

### Limitations

First, the variables on violence are self-reported and likely underestimate the real rate of violence. Certain groups may underreport more, for example consanguineously married women may report less violence due to fear of upsetting tightly knit kin groups. Similarly, richer women may also be less willing to report violence, perhaps due to increased social stigma due to higher pressure to maintain an appearance of high-status, or due to risk of losing access to wealth. Second, the data are cross-sectional in nature, results are correlational and causal inferences cannot be drawn. Third, we interpret natal family violence as a proxy of parent–offspring or sibling conflict over marriage, but it is unknown to what degree the violence is related to this conflict. Fourth, patrilocality is assumed but not measured. Whilst historically post-marital residence was patrilocal, nowadays most Jordanians live in nuclear households [50], although it is likely a fair assumption that couples still tend to live closer to, or in the same area as, the husband’s family. Indeed, when couples do live in the same building or house as extended family it is with the husband’s [50].

### Implications

Taking an evolutionary approach theorises domestic violence in terms of the different costs and benefits associated with marriage and reproduction to different family members, and hence how conflicts of interest within the family may arise. This approach helps to frame our understanding of different risk factors that are associated with violence from husbands and natal family VAW and girls. Violence from husbands is associated with determinants of fertility, including age and poverty, as well as the extent to which women are supported by their kin; whereas violence from natal family members is more associated with different kin-based marriage rules, where kin-based marriage may be associated with costs of inbreeding and benefits of families ties and inheritance rules, but where costs associated with attempts to violate kinship norms may often be associated with honour-related violence.

## SUPPLEMENTARY DATA

Supplementary data is available at *EMPH* online.

## Supplementary Material

eoac019_Supplementary_Data
