# Supplementary material for: Different predictors of intimate partner and natal family violence against women
Source: Evol Med Public Health. 2022 May 2;10(1):231–42. doi: 10.1093/emph/eoac019 (PMC9154062; doi:10.1093/emph/eoac019)
Supplement: eoac019_Supplementary_Data [file eoac019_supplementary_data.docx]

**Supplementary materials: “Different predictors of intimate partner and natal family violence against women”**

**Olympia L K Campbell & Ruth Mace**

**Figures**

**
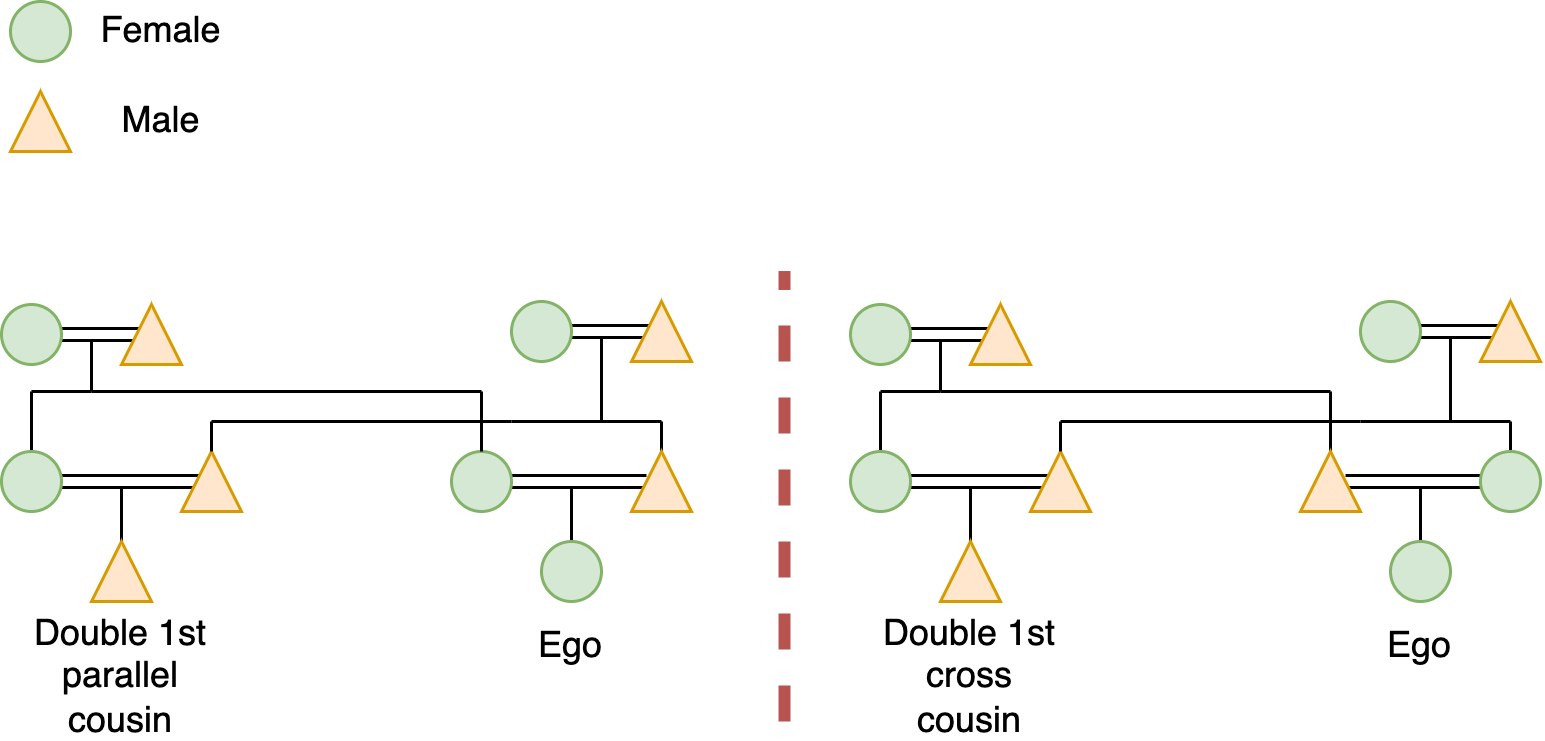
Figure S1**:

Figure S1: Type of double 1^st^ cousin in relation to ego. Double 1^st^ parallel cousins are related via both their father’s brother and their mother’s sister and result from two same sex siblings marrying another set of same sex siblings. Double 1^st^ cross cousins are related via both their father’s sister and their mother’s brother and result from two opposite sex siblings marrying another set of opposite sex siblings.

**Figure S2:**


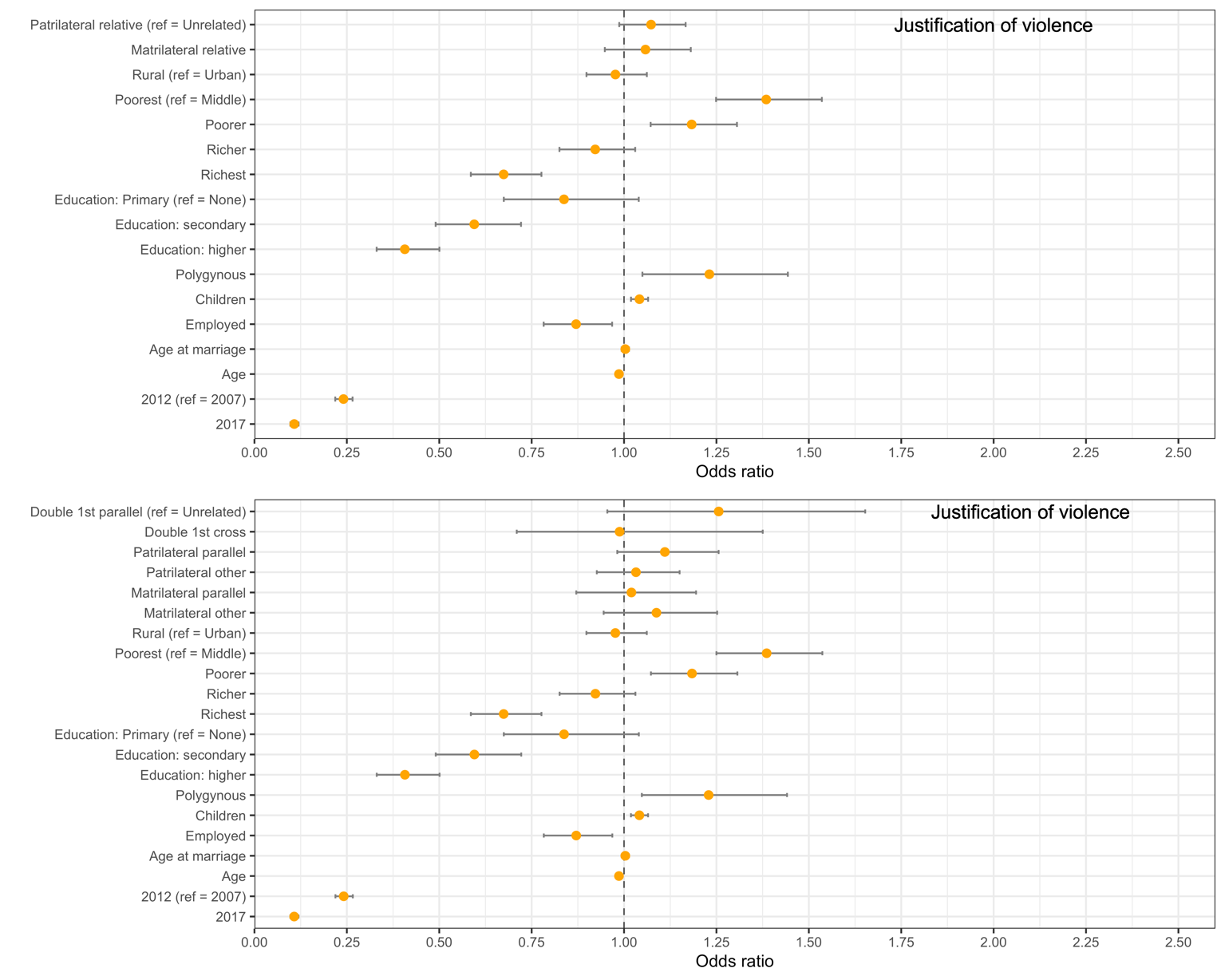


Figure S2: Odds ratios from multi-level logistic regressions showing likelihood of justifying violence from a husband. Top plot breaks consanguinity down into patrilateral and matrilateral relatives and the bottom plot breaks consanguinity down further into its constituent types

**Figure S3:**

**
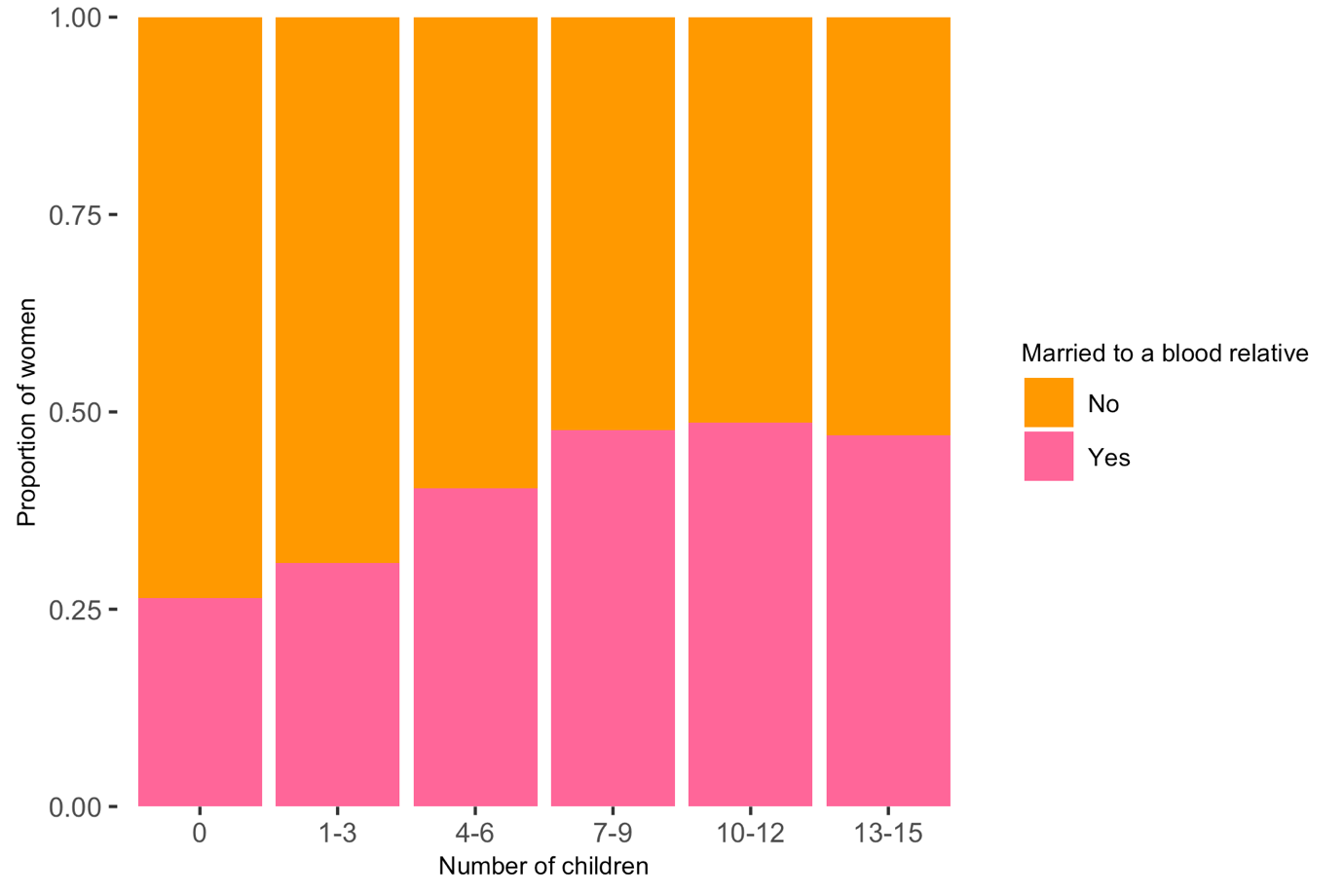
**

Figure S3: Proportion of women who are married to a blood relative grouped by the number of children they have

**Tables**

**Table S1**

|  | Model A: Violence from husband  OR (95% CI) | Model B: Violence from natal family  OR (95% CI) | Model C: Justification of violence from husband  OR (95% CI) |
| --- | --- | --- | --- |
|  | Models are controlled for survey year, age at marriage, age, polygyny, education, wealth, urban/rural living, number of children, and employment | | |
| Ref: Unrelated  Double 1^st^ parallel cousin | 0.67*  (0.45-0.98) | 0.84  (0.54-1.40) | 1.26  (0.95-1.65) |
| Double 1^st^ cross cousin | 0.75  (0.48-1.16) | 0.86  (0.52-1.42) | 0.99  (0.71-1.38) |
| Patrilateral parallel | 0.83*  (0.71-0.97) | 1.00  (0.84-1.19) | 1.11  (0.98-1.26) |
| Patrilateral other | 0.92  (0.80-1.05) | 0.95  (0.81-1.11) | 1.03  (0.93-1.15) |
| Matrilateral parallel | 0.87  (0.71-1.06) | 0.81  (0.64-1.02) | 1.02  (0.87-1.19) |
| Matrilateral other | 0.97  (0.82-1.16) | 0.84  (0.68-1.04) | 1.09  (0.94-1.25) |

Table S2: Odds ratios (OR) and confidence intervals (CI) of multi-level logistic regressions examining associations between each constituent type of cousin marriage and violence. Model A considers the likelihood of reporting violence from a husband, Model B the likelihood of reporting violence from a natal family member. *p<0.05, **p<0.01, ***p<0.001

**Table S2**

|  | Justification of violence from husband  OR (95% CI) |
| --- | --- |
| Ref: Unrelated  Patrilateral relative | 1.07  (0.99-1.17) |
| Matrilateral relative | 1.06  (0.95-1.18) |
| Ref: Urban  Rural | 0.98  (0.90-1.06) |
| Ref: Middle  Poorest | 1.38***  (1.25-1.54) |
| Poorer | 1.18***  (1.07-1.31) |
| Richer | 0.92  (0.83-1.03) |
| Richest | 0.67***  (0.59-0.78) |
| Ref: No education  Primary | 0.84  (0.67-1.04) |
| Secondary | 0.59***  (0.49-0.72) |
| Higher | 0.41***  (0.33-0.50) |
| Polygynous | 1.23*  (1.05-1.44) |
| Children | 1.04***  (1.02-1.07) |
| Employed | 0.87*  (0.78-0.97) |
| Age at marriage | 1.00  (0.99-1.01) |
| Age | 0.99***  (0.98-0.99) |
| Ref: 2007  2012 | 0.24***  (0.22-0.27) |
| 2017 | 0.11***  (0.10-0.12) |

Table S2: Odds ratios (OR) and confidence intervals (CI) of multi-level logistic regressions considering the likelihood of justifying violence from a husband *p<0.05, **p<0.01, ***p<0.001

**Table S3**

|  | Model A: Violence from husband  OR (95% CI) | Model B: Violence from natal family  OR (95% CI) |
| --- | --- | --- |
|  | Models are controlled for age at marriage, age, polygyny, education, wealth, urban/rural living, number of children, and employment | |
| Ref: Unrelated  Double 1^st^ parallel cousin (D1P) | 0.54  (0.25-1.14) | 1.16  (0.64-2.13) |
| Double 1^st^ cross cousin  (D1C) | 0.90  (0.41- 1.98) | 0.57  (0.22-1.45) |
| Patrilateral parallel  (PP) | 0.68*  (0.49-0.93) | 0.93  (0.67-1.27) |
| Patrilateral other  (PO) | 1.03  (0.79-1.32) | 1.01  (0.77-1.33) |
| Matrilateral parallel  (MP) | 0.82  (0.54-1.25) | 1.00  (0.65-1.53) |
| Matrilateral other  (MO) | 0.87  (0.61-1.23) | 1.11  (0.78-1.58) |
| Ref: 2007  2012 | 1.05  (0.91-1.20) | 0.99  (0.85-1.14) |
| 2017 | 0.65***  (0.56-0.76) | 0.25***  (0.21-0.30) |
| D1P X 2012 | 2.04  (0.72-5.84) | 0.43  (0.13-1.41) |
| D1C X 2012 | 0.84  (0.28-2.49) | 1.75  (0.52-5.90) |
| PP X 2012 | 1.19  (0.80-1.76) | 0.95  (0.64-1.41) |
| PO X 2012 | 0.78  (0.57-1.08) | 0.84  (0.60-1.19) |
| MP X 2012 | 1.06  (0.64-1.75) | 0.71  (0.42-1.20) |
| M0 X 2012 | 0.91  (0.59-1.42) | 0.66  0.42-1.03) |
| D1P X 2017 | 1.11  (0.43-2.88) | 0.63  (0.21-1.87) |
| D1C X 2017 | 0.72  (0.24-2.14) | 2.18  (0.58-8.14) |
| PP X 2017 | 1.54*  (1.01-2.35) | 1.82*  (1.11-2.97) |
| PO X 2017 | 0.99  (0.69-1.42) | 1.21  (0.75-1.94) |
| MP X 2017 | 1.06  (0.59-1.91) | 0.88  (0.39-1.97) |
| M0 X 2017 | 1.69*  (1.06-2.69) | 0.60  (0.20-1.26) |

Table S3: Odds ratios (OR) and confidence intervals (CI) of multi-level logistic regressions with interaction terms between consanguinity type and survey year. Model A considers the likelihood of reporting violence from a husband, Model B the likelihood of reporting violence from a natal family member. *p<0.05, **p<0.01, ***p<0.001

**Table S4**

|  | Model A: Violence from husband  OR (95% CI) | Model B: Violence from natal family  OR (95% CI) |
| --- | --- | --- |
| Ref: Unrelated  Patrilateral cousin | 0.93  (0.85-1.03) | 0.96  (0.86-1.08) |
| Matrilateral cousin | 0.90  (0.78-1.02) | 0.81**  (0.69-0.95) |

**Table S5**

|  | Model A: Violence from husband  OR (95% CI) | Model B: Violence from natal family  OR (95% CI) |
| --- | --- | --- |
| Ref: Unrelated  Double 1^st^ parallel cousin (D1P) | 0.73  (0.52-1.03) | 0.85  (0.56-1.30) |
| Double 1^st^ cross cousin  (D1C) | 0.71  (0.47-1.09) | 0.88  (0.54-1.43) |
| Patrilateral parallel  (PP) | 0.92  (0.80-1.07) | 1.01  (0.85-1.19) |
| Patrilateral other  (PO) | 0.99  (0.87-1.12) | 0.95  (0.82-1.10) |
| Matrilateral parallel  (MP) | 0.86  (0.71-1.04) | 0.80  (0.64-1.01) |
| Matrilateral other  (MO) | 0.93  (0.79-1.09) | 0.82*  (0.67-1.00) |

Table S4 & 5: Odds ratios (OR) and confidence intervals (CI) of multi-level logistic regressions with random intercepts for survey year. Model A considers the likelihood of reporting violence from a husband, Model B the likelihood of reporting violence from a natal family member. *p<0.05, **p<0.01, ***p<0.001
